# Cardiac Mortality Among 200 000 Five-Year Survivors of Cancer Diagnosed at 15 to 39 Years of Age: The Teenage and Young Adult Cancer Survivor Study

**DOI:** 10.1161/CIRCULATIONAHA.116.022514

**Published:** 2016-11-14

**Authors:** Katherine E. Henson, Raoul C. Reulen, David L. Winter, Chloe J. Bright, Miranda M. Fidler, Clare Frobisher, Joyeeta Guha, Kwok F. Wong, Julie Kelly, Angela B. Edgar, Martin G. McCabe, Jeremy Whelan, David J. Cutter, Sarah C. Darby, Mike M. Hawkins

**Affiliations:** From Clinical Trial Service Unit, Nuffield Department of Population Health, University of Oxford, United Kingdom (K.E.H., D.J.C., S.C.D.); Centre for Childhood Cancer Survivor Studies, Institute of Applied Health Research, University of Birmingham, Edgbaston, United Kingdom (K.E.H., R.C.R., D.L.W., C.J.B., M.M.F., C.F., J.G., K.F.W., J.K., M.M.H.); Department of Paediatric Haematology and Oncology, Royal Hospital for Sick Children, University of Edinburgh, United Kingdom (A.B.E.); Institute of Cancer Sciences, University of Manchester, Manchester Academic Health Science Centre (M.C.M.); National Institute for Health Research University College London Hospitals Biomedical Research Centre, United Kingdom (J.W.); and British Heart Foundation Centre for Research Excellence (D.J.C, S.C.D).

**Keywords:** adolescent, cardiac deaths, epidemiology, heart diseases, mortality, neoplasms

## Abstract

**Background:**

Survivors of teenage and young adult cancer are acknowledged as understudied. Little is known about their long-term adverse health risks, particularly of cardiac disease that is increased in other cancer populations where cardiotoxic treatments have been used.

**Methods:**

The Teenage and Young Adult Cancer Survivor Study cohort comprises 200 945 5-year survivors of cancer diagnosed at 15 to 39 years of age in England and Wales from 1971 to 2006, and followed to 2014. Standardized mortality ratios, absolute excess risks, and cumulative risks were calculated.

**Results:**

Two thousand sixteen survivors died of cardiac disease. For all cancers combined, the standardized mortality ratios for all cardiac diseases combined was greatest for individuals diagnosed at 15 to 19 years of age (4.2; 95% confidence interval, 3.4–5.2) decreasing to 1.2 (95% confidence interval, 1.1–1.3) for individuals aged 35 to 39 years (2*P* for trend <0.0001). Similar patterns were observed for both standardized mortality ratios and absolute excess risks for ischemic heart disease, valvular heart disease, and cardiomyopathy. Survivors of Hodgkin lymphoma, acute myeloid leukaemia, genitourinary cancers other than bladder cancer, non-Hodgkin lymphoma, lung cancer, leukaemia other than acute myeloid, central nervous system tumour, cervical cancer, and breast cancer experienced 3.8, 2.7, 2.0, 1.7, 1.7, 1.6, 1.4, 1.3 and 1.2 times the number of cardiac deaths expected from the general population, respectively. Among survivors of Hodgkin lymphoma aged over 60 years, almost 30% of the total excess number of deaths observed were due to heart disease.

**Conclusions:**

This study of over 200 000 cancer survivors shows that age at cancer diagnosis was critical in determining subsequent cardiac mortality risk. For the first time, risk estimates of cardiac death after each cancer diagnosed between the ages of 15 and 39 years have been derived from a large population-based cohort with prolonged follow-up. The evidence here provides an initial basis for developing evidence-based follow-up guidelines.

## Abstract 2

Supplemental Digital Content is available in the text.

Survivors of cancer diagnosed in teenagers or young adults are internationally acknowledged as an understudied population.^1^ The majority of survivorship research has focused on survivors of specific cancers of adulthood or on childhood cancers.^2–4^ Findings from studies of adult or childhood cancer survivor populations may not necessarily be directly extrapolated to teenagers and young adults because the tumors diagnosed are distinct in terms of the types of cancers diagnosed, hormonal factors (eg, puberty), tumor biology, and the developmental maturity of the organs at highest risk of toxicity.^5–8^ The historical and ongoing paucity of generally accepted teenage and young adult (TYA) cancer treatment protocols means that individuals may have received either adult or pediatric treatment protocols, which may be very different in their approach and treatment intensity depending on the cancer type, treatment center, and clinician responsible for their care. Thus, it is necessary to assess TYA cancer survivors as a distinct group.

Cardiac disease has been found to be the leading cause of treatment-related nonneoplastic death among survivors of childhood cancer,^9,10^ breast cancer,^11,12^ and Hodgkin lymphoma.^13,14^ As yet, the risk of cardiac mortality has not been investigated comprehensively within a large population of TYA cancer survivors. Only 2 studies have analyzed cause-specific mortality within an entire population of TYA cancer survivors,^15,16^ but relatively modest cohort sizes (n=9245 and n=16 769) have left important questions unanswered, particularly regarding the mortality risk from cardiac disease and how this risk may vary by cancer type. There is clearly a need for estimates of cardiac mortality risks among such subgroups of survivors of TYA cancer.

Studies of hospitalization complement the understanding of mortality risk in a population. Recently, a number of studies have addressed the risk of hospitalization from cardiovascular disease among survivors of cancer diagnosed before 40 years of age.^17–20^ The largest of these was performed in Denmark using 43 153 cancer survivors^17^ but provided limited information on cardiac disease specifically.

The objective of this large-scale population-based study was to investigate the long-term risk of cardiac mortality among 5-year survivors of TYA cancer. To our knowledge, this cohort is the largest ever assembled in relation to this age range, with >200 000 5-year survivors and 2.8 million person-years of follow-up, 15% of whom were followed for >30 years. This study includes >7 times more cancer survivors than the 2 previous studies combined.

## Methods

### The Teenage and Young Adult Cancer Survivor Study

TYACSS (The Teenage and Young Adult Cancer Survivor Study) is a population-based cohort comprising 200 945 individuals with cancer diagnosed at 15 to 39 years of age, in England and Wales, between 1971 and 2006 inclusive who survived at least 5 years from diagnosis. Individuals had to be diagnosed with a malignant tumor, unless diagnosed with tumors of the brain or bladder, in which case all malignant, benign, and unspecified tumors were included. All individuals diagnosed with a first primary cancer satisfying these criteria were identified and included (see Table 1). Any subsequent primary cancers in these individuals were identified and excluded. Comparison with the British Childhood Cancer Survivor Study^21^ enabled identification and exclusion of individuals who were previously diagnosed with cancer before 15 years of age. Legal consent to process patient-identifiable information was obtained from the Confidentiality Advisory Group (NIGB: ECC 3–04 (c) / 2010). Ethical approval was given by the National Research Ethics Committee (ref: 16/LO/0895).

**Table 1.**
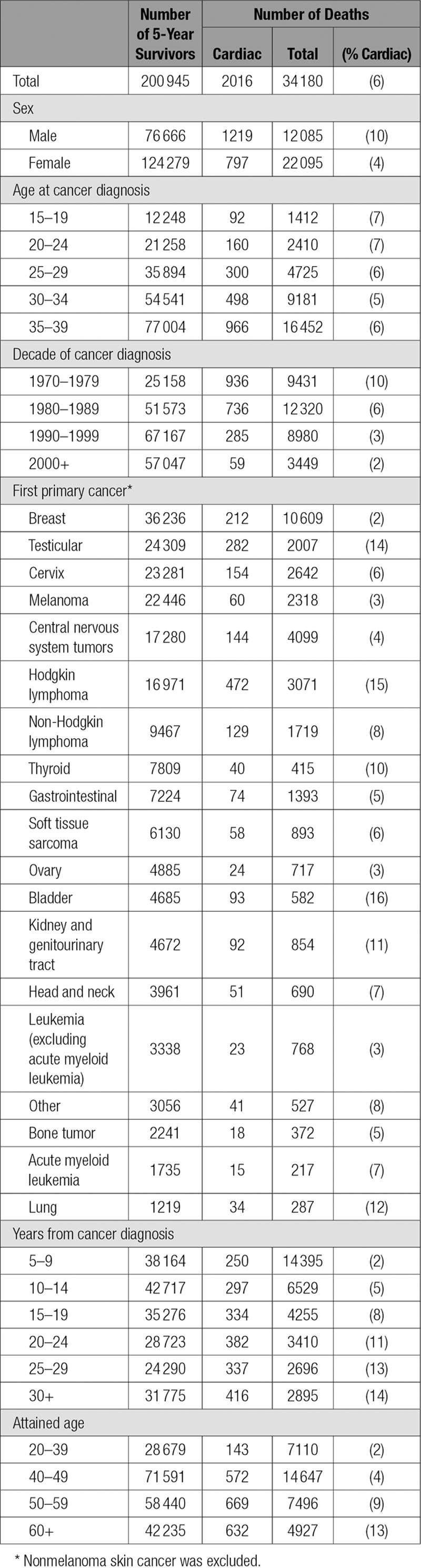
Patient Characteristics of Teenage and Young Adult Cancer Survivor Study

The cohort was ascertained through the Office of National Statistics in England and the Welsh Cancer Registry. Linkage by the Health and Social Care Information Center provided the vital status and emigration status for each survivor. For all deaths, the underlying cause of death was also sought from the Health and Social Care Information Centre, coded by using the *International Classification of Diseases*, *Ninth* or *Tenth Revision*, applicable to the year of death. The cause of death was available for 98.4% of deaths.

Cancer groupings were based on the internationally established classification scheme for TYA cancers^22,23^ that was slightly modified to create finer groupings (see online-only Data Supplement Table I).

Cardiac disease was defined using *International Classification of Diseases*, *Tenth Revision* codes: I01, I02.0, I05 to I09, I11, I13, I20 to I25, I27.1 to I27.9, and I30 to I52 and the corresponding *International Classification of Diseases*, *Ninth* *Revision* codes: 391, 392.0, 393 to 398, 402, 404, 410 to 414, 416, and 420 to 429. A mutually exclusive and exhaustive classification of the *International Classification of Diseases* codes was performed by a clinician (D.J.C.) to define cardiac disease subtypes (see online-only Data Supplement Table II).

### Statistical Analysis

Each individual’s contribution to the person-years at risk began at the date of 5-year survival and ended at the earliest of February 28, 2014, death, or loss to follow-up because of emigration.

Standardized mortality ratios (SMRs) and absolute excess risks (AERs) were calculated by using standard cohort techniques.^24^ Population-based expected numbers of deaths were derived from age (5-year groups), sex, and calendar-year (1-year groups) specific death rates for England and Wales combined. SMRs were calculated as the ratio of the observed to expected numbers of deaths. AERs were calculated as the observed number of deaths minus the expected number, divided by the person-years at risk, and this quotient was multiplied by 10 000.

Tests for trend and heterogeneity were performed using likelihood ratio tests based on Poisson regression models. Statistical significance was defined as 2*P*<0.05.

Multivariable Poisson regression models for the SMR and AER were used to evaluate the potential confounding effect of specified demographic and cancer-related factors.^24^ If the results from univariable (Tables 2 through 5) and multivariable (online-only Data Supplement Tables III and IV) modeling were similar, that is, there was no evidence of confounding, then the findings in the text of Results and Discussion were reported in terms of SMRs and AERs. If the univariable and multivariable modeling results were not similar, that is, there was evidence of confounding, then the multivariable results were reported in the text of Results and Discussion in terms of relative risks or excess mortality ratios. Relative risks and excess mortality ratios can be interpreted as ratios of SMRs and AERs, respectively, adjusted for other factors included within the model. The factors investigated were sex, age at cancer diagnosis (5-year groups), decade of cancer diagnosis, time since diagnosis (5-year groups), attained age (20–39, 40–49, 50–59, and 60+ years), and first primary cancer type. Attained age and time since diagnosis were never fitted in the same multivariable model because of strong collinearity. Within Tables 2, 3, 4, and 5, in addition to providing the *P* value from the univariable modeling for each factor, we also provide the *P* value from the multivariable modeling, which is reported in online-only Data Supplement Tables III and IV. This enables the reader to assess whether a statistically significant relationship remains after adjustment for the specified confounders. All cancers were analyzed together, and the specific cancers with at least 100 cardiac deaths and a significantly elevated SMR were investigated separately with both univariable and multivariable Poisson regression.

**Table 2.**
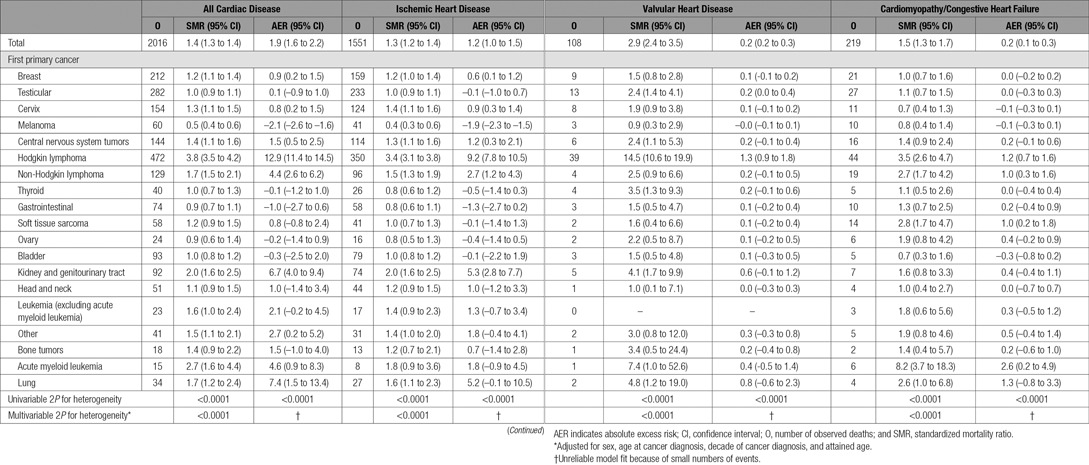
SMRs and AERs per 10 000 Person-Years at Risk According to First Primary Cancer for All Cardiac Disease Combined, Ischemic Heart Disease, Valvular Heart Disease, and Cardiomyopathy/Congestive Heart Failure

**Table 3.**
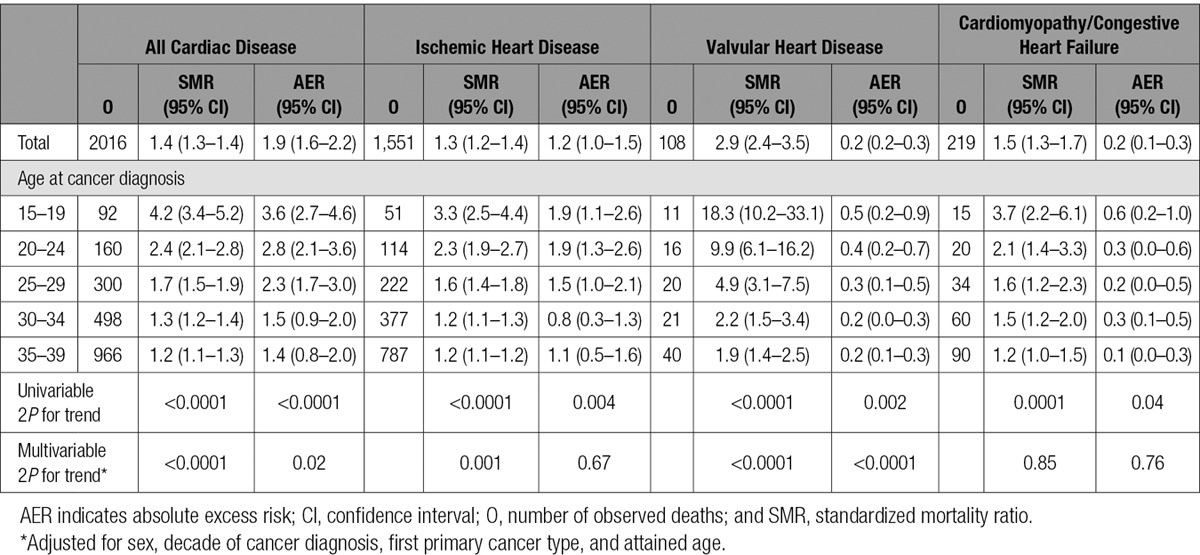
SMRs and AERs per 10 000 Person-Years at Risk According to Age at Cancer Diagnosis, for All Cardiac Disease Combined, Ischemic Heart Disease, Valvular Heart Disease, and Cardiomyopathy/Congestive Heart Failure

**Table 4.**
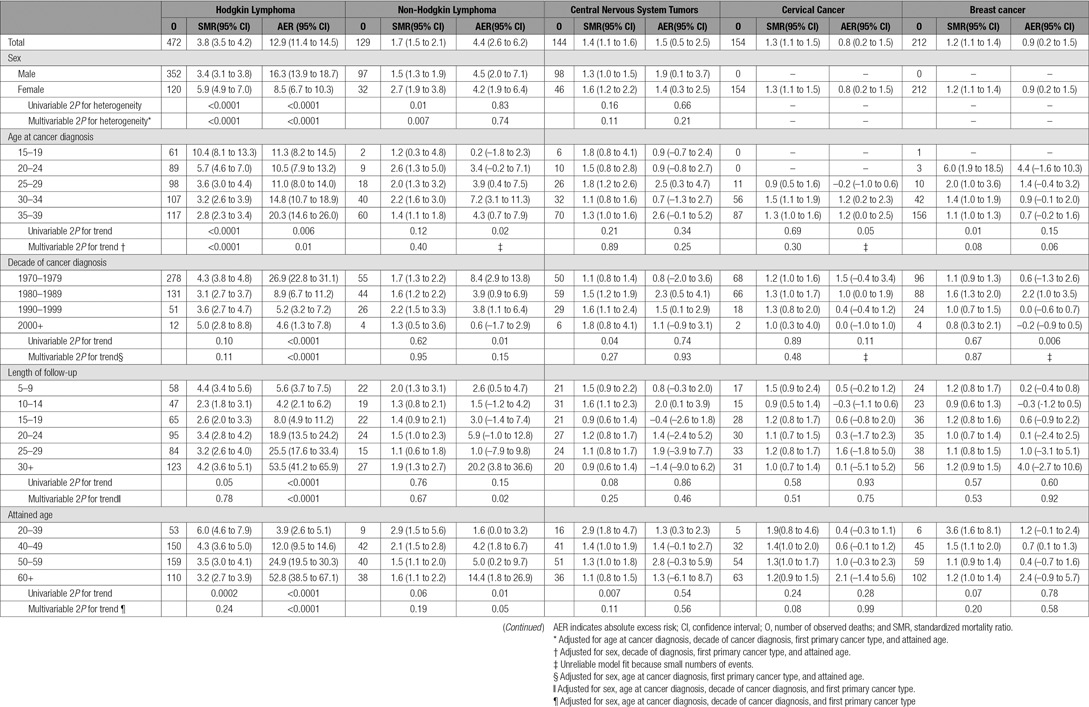
SMRs and AERs per 10 000 Person-Years at Risk According to Sex, Age at Cancer Diagnosis, Decade of Cancer Diagnosis, Years Since Cancer Diagnosis and Attained Age for All Cardiac Disease Combined for First Primary Cancers With at Least 100 Cardiac Deaths and a Significantly Elevated SMR

**Table 5.**
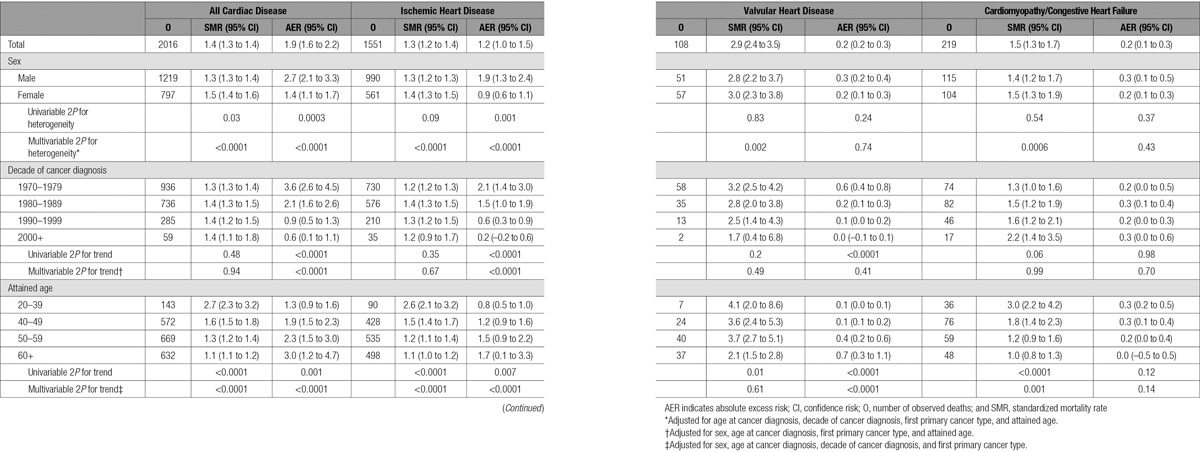
SMRs and AERs per 10 000 Person-Years at Risk According to Sex, Decade of Cancer Diagnosis, and Attained Age for All Cardiac Disease Combined, Ischemic Heart Disease, Valvular Heart Disease, and Cardiomyopathy/Congestive Heart Failure

Cumulative risk of mortality, taking account of competing risk of death from any cause other than cardiac disease, was estimated.

All calculations used Stata 13.^25^

## Results

The cohort contributed a total of 2 867 879 person-years at risk with a mean follow-up from 5 years after diagnosis of cancer of 14.3 years. By the end of February 2014, 34 180 (17%) individuals had died: 2016 (6%) deaths were attributable to cardiac disease (see Table 1). The subtypes of cardiac death were: 1551 (77%) ischemic heart disease (IHD), 219 (11%) cardiomyopathy or congestive heart failure (CM/HF), 108 (5%) valvular heart disease (VHD), 38 (2%) rheumatic valvular heart disease, 22 (1%) arrhythmias, 18 (1%) pericardial heart disease, and 60 (3%) deaths attributable to other cardiac causes.

### Cardiac Mortality Risk for All Cancers Combined

The SMR for all types of cardiac disease combined was 1.4 (95% confidence interval [CI], 1.3–1.4) and the AER per 10 000 person-years was 1.9 (95% CI, 1.6–2.2) (Table 2). Cardiac deaths accounted for 2% of all excess deaths, and the proportion attributable to cardiac disease increased slightly with attained age, contributing 1% among ages 20 to 39 in comparison with 4% at age 60+ (online-only Data Supplement Table V). The SMR for IHD was 1.3 (95% CI, 1.2–1.4) and the AER per 10 000 person-years was 1.2 (95% CI, 1.0–1.5) (Table 2).

### Variation in Cardiac Mortality Risk by First Primary Cancer

There was strong evidence of heterogeneity across the cancer types for both SMRs and AERs (2*P*<0.0001). The highest SMR and AER for cardiac mortality were observed after Hodgkin lymphoma (SMR, 3.8; 95% CI, 3.5–4.2; AER, 12.9; 95% CI, 11.4–14.5; see Table 2). The other first primary cancer groups with a significantly raised SMR for cardiac mortality were acute myeloid leukemia (2.7; 95% CI, 1.6–4.4), genitourinary cancers other than bladder cancer (2.0; 95% CI, 1.6–2.5), lung cancer (1.7; 95% CI, 1.2–2.4), non-Hodgkin lymphoma (1.7; 95% CI, 1.5–2.1), leukemia (excluding acute myeloid leukemia) (1.6, 95% CI, 1.0–2.4), central nervous system tumors (1.4; 95% CI, 1.1–1.6), cervical cancer (1.3; 95% CI, 1.1–1.5), and breast cancer (1.2; 95% CI, 1.1–1.4).

When different cancer types were considered, there were considerable variations in the extent to which the different types of cardiac disease were increased. Hodgkin lymphoma survivors experienced the highest SMR and AER for IHD and VHD. However, analyses of CM/HF mortality indicated that acute myeloid leukemia survivors had the highest SMR (8.2; 95% CI, 3.7–18.3) and highest AER (2.6; 95% CI, 0.2–4.9).

### Variation in Cardiac Mortality Risk by Age and Decade of Diagnosis

For all cancers combined, a highly significant decreasing trend (2*P*<0.0001) with increasing age at cancer diagnosis was shown for both SMRs and AERs for all cardiac disease (Table 3). The SMR was greatest for individuals diagnosed with cancer at 15 to 19 years of age (4.2; 95% CI, 3.4–5.2), and decreased to 1.2 (95% CI, 1.1–1.3) among individuals diagnosed with cancer at 35 to 39 years of age. The AER declined from 3.6 (95% CI, 2.7–4.6) to 1.4 (95% CI, 0.8–2.0) following diagnosis at ages 15 to 19 and 35 to 39, respectively. These trends with age at cancer diagnosis were consistent across IHD, VHD, and CM/HF mortality (all SMR 2*P*≤0.0001 and AER 2*P*≤0.04). There was a substantially raised SMR for valvular heart disease among individuals diagnosed with cancer at 15 to 19 years of age (SMR, 18.3; 95% CI, 10.2–33.1). These relationships with age at diagnosis remained after multivariable adjustment, apart from the excess mortality ratio for IHD and for both relative risk and excess mortality ratio for CM/HF (see online-only Data Supplement Table III).

The overall significant decreasing trend in SMRs of all cardiac disease with age at cancer diagnosis was primarily attributable to survivors of Hodgkin lymphoma and breast cancer (Table 4). For Hodgkin lymphoma, those diagnosed at 15 to 19 years of age had an SMR of 10.4 (95% CI, 8.1–13.3), in comparison with those diagnosed at 35 to 39 years of age with an SMR of 2.8 (95% CI, 2.3–3.4). The almost corresponding SMRs after breast cancer were 6.0 (95% CI, 1.9–18.5) and 1.1 (95% CI, 1.0–1.3) respectively.

As shown in Table 5, the AERs for all cardiac deaths, IHD deaths, and VHD deaths all increased significantly with attained age, and this remained after multivariable adjustment. However, the AERs for CM/HF deaths did not vary significantly by attained age (Table 5). The AERs for all cardiac deaths and IHD deaths declined with more recent decade of diagnosis, but AERs for VHD and CM/HF deaths did not vary significantly with decade of diagnosis (Table 5), taking into account the multivariable adjustment.

### Hodgkin Lymphoma Survivors

Among survivors of Hodgkin lymphoma aged 60+, 27.5% of all excess deaths were attributable to cardiac causes (online-only Data Supplement Table V). The cumulative risk of cardiac mortality was greatest for individuals diagnosed with Hodgkin lymphoma at a younger age: among those diagnosed at 15 to 19 years of age, by age 55 years the cumulative risk was 6.9% in comparison with 2.0% for those diagnosed at 35 to 39 years of age (log-rank 2*P*<0.0001). The corresponding expected cumulative mortality by age 55 was 0.9% (Figure).

**Figure.**
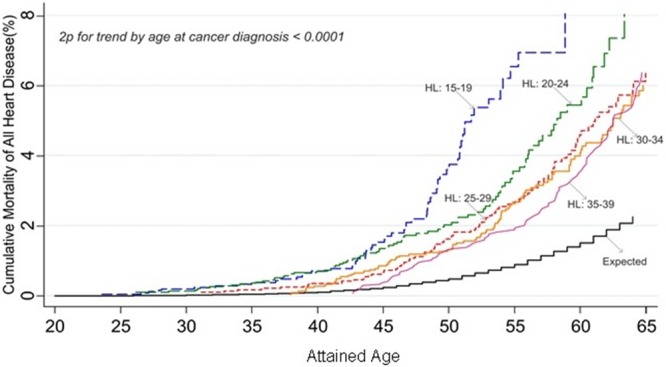
**Cardiac mortality according to attained age.** Cumulative mortality from cardiac disease among 5-year survivors of Hodgkin lymphoma according to attained age by age at cancer diagnosis. HL indicate Hodgkin lymphoma.

## Discussion

This largest ever study of >200 000 survivors of cancer diagnosed in teenagers and young adults reveals that age at diagnosis and type of cancer were important in determining risk of cardiac mortality. Although cancer treatments for specific cancer types have changed over the decades during which members of the cohort were treated, the variation in treatments for any specific cancer over the decades is, in general, appreciably less than the variation in treatments between different specific cancers; therefore, cancer type here may be regarded as an approximate surrogate for treatment history. This study provides evidence on which to base risk stratification for the clinical follow-up of survivors. Here, for the first time, risk estimates relating to the entire spectrum of cancers diagnosed between ages 15 and 39 have been investigated in the long term. This is important information for both clinicians and survivors.

Survivors of Hodgkin lymphoma were found to have the greatest SMR and AER for cardiac disease, and a strong decline in both the SMR and the cumulative risk with increasing age at diagnosis was clear among these survivors. The size of the cohort and extended age range enabled us to demonstrate the age effect more clearly than ever before. It was independent of attained age and years from diagnosis. A similar relationship was observed among survivors of breast cancer. However, the absolute excess number of cardiac deaths in breast cancer survivors diagnosed in the youngest age group was small.

### Variation in Cardiac Mortality Risk by Type of Cardiac Disease and Type and Decade of Cancer Diagnosis

The AER for cardiac deaths overall and for ischemic heart disease specifically declined with more recent decades of treatment, possibly because the net effect of changes in cancer treatments has resulted in a reduction in the overall risk of cardiotoxicity following treatment for cancer. However, it was somewhat surprising that the risk of cardiomyopathy did not vary with decade of treatment given the introduction of anthracyclines in 1980s and subsequent widespread use.

Ischemic heart disease, for which radiation is a known risk factor,^26^ accounted for 74%, 74%, and 75% of cardiac deaths after Hodgkin lymphoma, non-Hodgkin lymphoma, and breast cancer, respectively, and considering that external beam radiotherapy to the thorax would often have been an element of treatment for such cancers, it is likely that radiotherapy contributed to the excess risks observed. The excess deaths from valvular heart disease increased with increasing attained age, even after adjustment for specified confounding factors. Radiation-related valvular heart disease has been shown to have a long latency period,^27^ with 1 study of Hodgkin lymphoma survivors finding a median interval of 22 years between treatment and symptomatic cardiac disease.^28^ This finding may reflect older radiotherapy techniques and higher radiation doses in the earlier decades of diagnosis because valvular disease has been shown to be strongly related to dose to the heart.^29^ Anthracyclines have been shown to be cardiotoxic, with a recent meta-analysis of randomized controlled trials finding that anthracyclines were associated with a 5-fold increased risk of congestive heart failure in comparison with nonanthracycline regimens.^30^ The 8-fold increased risk of CM/HF after acute myeloid leukemia is likely because of anthracyclines, bearing in mind the widespread use of these drugs to treat this disease.

### Cardiac Risk Among TYA Cancer Survivors in Context

A study by the North American Childhood Cancer Survivor Study sought to evaluate the contribution of modifiable cardiovascular risk factors in addition to treatment-related cardiac damage.^31^ The authors concluded that “it is imperative that childhood cancer survivors exposed to chest-directed radiotherapy or anthracycline chemotherapy have regular blood pressure monitoring and appropriate management as a high-risk group.”^31^ A similar study is needed among survivors of TYA cancer.

The present study suggests that the SMRs for cardiac mortality among survivors of TYA cancers are lower than those seen in childhood cancer survivors, where increases for all cardiac diseases combined have ranged from 3.5-fold (95% CI, 2.9–3.2) in the British Childhood Cancer Survivor Study^9^ to 7-fold in the North American Childhood Cancer Survivor Study.^10^ Direct comparison of the results of these studies is, however, difficult because differences in treatment, in the demographics, and in the length of follow-up in the different cohorts could lead to substantial differences in risk. Studies pooling all cancer survivors diagnosed before 40 years of age would provide greater opportunities to control for such confounding influence.

### Strengths and Limitations

This is by far the largest population-based study yet to investigate cardiac mortality risk among survivors of cancer diagnosed in teenagers and young adults with a total of 2 867 878 person-years of follow-up. In this cohort, 28% (n=56 035) were followed up for at least 25 years from cancer diagnosis, 53% for between 10 and 25 years, and 19% for a maximum of 10 years. This extended follow-up included 1033 IHD, 107 CM/HF, and 77 VHD deaths after 50 years of age, providing a large number of events for analysis.

The main limitation is the lack of detailed information on exposure to the most relevant treatment modalities: radiotherapy and chemotherapy. Other conventional cardiovascular risk factors (eg, smoking) were also not available, and the laterality of breast cancer, a known determinant of cardiac dose of radiation, was too incomplete for analysis. Nested case-control studies will be needed to address dose-response relationships of risk in relation to treatment exposures and other risk factors, and will allow further study of how these relationships may vary, for example, with age at exposure.

### Conclusion

This study demonstrates for the first time that age at cancer diagnosis is important in determining the excess risk of cardiac death among an entire population of survivors of TYA cancer. This age at diagnosis effect was primarily accounted for by survivors of Hodgkin lymphoma and breast cancer, providing useful risk stratification evidence. For evidence-based clinical follow-up of survivors estimates of risks that are reliable (from large-scale studies) and unbiased (from population-based studies) are needed for specific groups of survivors defined in terms of cancer type, age at cancer, and type of treatment. Previously, this detailed information has been available, in part, for survivors of Hodgkin lymphoma,^13,29,32^ but has been lacking for most other TYA cancer types. Although not stratified by treatment, we provide risk estimates for each TYA cancer stratified by age at diagnosis, which is a considerable advance on previous knowledge.

## Appendix

Study collaborators include: Professor Sarah Darby, University of Oxford; Dr Angela Edgar, Royal Hospital for Sick Children, Edinburgh; Dr Richard Feltbower, University of Leeds; Dr Lorna Anne Fern, University College London; Dr Diana Greenfield, University of Sheffield; Dr Tony Moran, North West Cancer Intelligence Service; Professor John Radford, University of Manchester; Dr Peter Rose, University of Oxford; Dr Helen Alexandra Spoudeas, University College London Hospitals NHS Foundation Trust; Dr William Hamish Wallace, Royal Hospital for Sick Children, Edinburgh; Professor Jeremy Whelan, University College London Hospitals NHS (National Health Service) Foundation Trust London.

## Acknowledgments

The Teenage and Young Adult Cancer Survivor Study acknowledges and thanks its data providers: Office for National Statistics, Welsh Cancer Registry, and Health and Social Care Information Center.

We are thankful to the National Cancer Research Institute – Teenage and Young Adult Clinical Studies Group.

## Sources of Funding

This study would not have been possible without funding to the University of Birmingham from: Cancer Research UK (grant C386/A11709), and the National Institute for Health Research to whom we offer our profound thanks. Other Birmingham funding was through a postdoctoral fellowship to Dr Raoul Reulen from the National Institute for Health Research (PDF-2012-05-280).

This work was carried out as part of Katherine Henson’s doctoral work at the University of Oxford. Katherine was funded by a research contract to the University of Oxford under the Department of Health Policy Research Program (Studies of Ionising Radiation and the Risk of Heart Disease, 091/0203) and by a studentship from the Nuffield Department of Population Health. Other Oxford funding was provided by Cancer Research UK (grant C8225/A21133) and by core funding to the Clinical Trial Service Unit (from Cancer Research UK, Medical Research Council, British Heart Foundation) and by the British Heart Foundation Center for Research Excellence (grant no RE/13/1/30181).

## Disclosures

All authors declare that they have no conflicts of interest in relation to this work. This report is independent research and the views expressed in this publication are those of the author(s) and not necessarily those of the NHS, the National Institute for Health Research, or the Department of Health.

## Supplementary Material

## Clinical Perspective

### What Is New?

- Few studies to date have investigated the long-term adverse effects of cancer treatment among survivors of cancer diagnosed in teenagers and young adults; this study is the largest to date and is population based with lengthy follow-up.
- It provides estimates of the risks of death from heart disease for each individual type of cancer.
- It also provides estimates of the risk of individual cardiac diseases, with special emphasis on the dependence of the risk on the age at which the initial cancer was diagnosed.
- The results will enable those who are likely to be most at risk to be identified.

### What Are the Clinical Implications?

- This study provides insight into the cardiotoxicity of the treatments given in the past to teenagers and young adults for each individual type of cancer.
- It shows that age at cancer diagnosis is an important determinant of the risk of death from heart disease many decades later.
- This age effect was most apparent for survivors of Hodgkin lymphoma, who were also found to be at the greatest risk overall.
- Population-based risk estimates for survivors of individual types of cancer are crucial for the development of evidence-based clinical follow-up guidelines and recommendations, as well as for future screening recommendations.
