# Supplementary material for: Cardiac Mortality Among 200 000 Five-Year Survivors of Cancer Diagnosed at 15 to 39 Years of Age: The Teenage and Young Adult Cancer Survivor Study
Source: Circulation. 2016 Nov 14;134(20):1519–31. doi: 10.1161/CIRCULATIONAHA.116.022514 (PMC5106083; doi:10.1161/CIRCULATIONAHA.116.022514)
Supplement: Supplementary file 1 [file cir-134-1519-s001.pdf]

# SUPPLEMENTAL MATERIAL

## Cardiac mortality among 200,000 five-year survivors of cancer diagnosed aged 15-39 years: The Teenage and Young Adult Cancer Survivor Study

Henson, Cardiac mortality among teenagers and young adults

Katherine E Henson DPhil [1,2], Raoul C Reulen PhD [2], David L Winter HNC [2], Chloe J Bright MSc [2], Miranda M Fidler PhD [2], Clare Frobisher PhD [2], Joyeeta Guha PhD [2], Kwok F Wong PhD [2], Julie Kelly [2], Angela B Edgar MD[3], Martin G McCabe PhD [4], Jeremy Whelan MD FRCP MBBS [5], David J Cutter DPhil [1], Sarah C Darby DPhil [1], Mike M Hawkins DPhil [2]

[1] Clinical Trial Service Unit, Nuffield Department of Population Health, University of Oxford, Richard Doll Building, Old Road Campus, Oxford

[2]Centre for Childhood Cancer Survivor Studies, Institute of Applied Health Research, Public Health Building, University of Birmingham, Edgbaston, Birmingham

[3] Department of Paediatric Haematology and Oncology, Royal Hospital for Sick Children, University of Edinburgh, Edinburgh EH9 1LF

[4] Institute of Cancer Sciences, University of Manchester, Manchester Academic Health Science Centre

[5] National Institute for Health Research University College London Hospitals Biomedical Research Centre, London

Corresponding author: Professor Mike Hawkins

Centre for Childhood Cancer Survivor Studies, Institute of Applied Health Research, Public Health Building, University of Birmingham, Birmingham, B15 2TT;

+44 (0)121 414 7924; m.m.hawkins@bham.ac.uk

Disclaimers: All authors declare that they have no conflicts of interest in relation to this work.

Subject Codes: Mortality/Survival, Epidemiology, Risk Factors

## Supplemental Table 1: First Primary Cancer Classification Detail –Modified from Birch et al [1]

| First Primary Cancer Grouping | Specific Cancer Description |
|---|---|
| Breast | Breast |
| | Germ cell gonadal |
| | Other specified gonadal tumours |
| Testicular | Testicular |
| Cervix | Cervix |
| Melanoma | Melanoma & Naevi |
| | Pilocytic astrocytoma |
| | Other specified astrocytoma |
| | Glioblastoma/anaplastic astrocytoma |
| | Astrocytoma NOS [*] |
| | Oligodendroglioma |
| | Other specified glioma |
| | Glioma, NOS |
| | Ependymoma |
| | Medulloblastoma |
| | Supratentorial PNET |
| | Craniopharyngioma |
| | Other Pituitary tumours |
| | Pineal tumours |
| | Choroid plexus tumours |
| | Meningioma |
| | CNS nerve sheath tumours |
| | Other specified CNS [†] |
| | Unspecified malignant CNS |
| | Unspecified benign CNS |
| Central Nervous System Tumours | Other CNS |
| | Germ cell intracranial |
| | Hodgkin Disease (specified) |
| Hodgkin | Hodgkin Disease NOS |
| | Specified NHL [‡] |
| | Unspecified NHL |
| Non-Hodgkin Lymphoma | Misc lymphoreticular neops NEC |
| Thyroid | Thyroid |
| | Colon & rectum |
| | Stomach |
| | Liver |
| | Pancreas |
| Gastrointestinal | Gastrointesinal tract (other) |
| | Fibrosarcoma |
| | Malig fibrous histiocytoma |
| | Dermatofibrosarcoma |
| | Rhabdomyosarcoma |
| | Liposarcoma |
| | Leiomyosarcoma |
| | Synovial sarcoma |
| | Clear cell sarcoma |
| | Blood vessel tumours |
| | Nerve sheath tumours |
| | Alveolar soft part sarcoma |
| | Other Specified Soft Tissue Sarcoma |
| Soft Tissue Sarcoma | Unspecified Soft Tissue Sarcoma |
| | Ovary |
| | Germ cell gonadal (if female) |
| Ovary | Other specified gonadal tumours(if female) |
| | Bladder |
| Bladder | Other bladder |
| | GU tract [§] |
| | Kidney |
| Kidney and GU tract [§] | GU tract (other) |

| | |
|---|---|
| | Wilms tumour |
| | Nasopharyngeal |
| | Other lip/oral cavity/pharynx |
| Head & Neck | Other Nasal cavity/middle ear |
| | Acute Lymphoid Leukaemia |
| | Chronic Myeloid Leukaemia |
| | Other Lymphoid Leukaemia |
| | Other Myeloid Leukaemia |
| | Other Specified Leukaemia |
| Leukaemia (excl. AML[‖]) | Other Unspecified Leukaemia |
| | Osteosarcoma |
| | Chondrosarcoma |
| | Ewing sarcoma |
| | Ewing sarcoma NOT bone |
| | Ewing sarcoma site unspecified |
| | Other bone tumours specific |
| Bone Tumour | Bone tumours unspecified |
| Acute Myeloid Leukaemia | Acute Myeloid Leukaemia |
| Lung | Trachea, bronchus & lung |

[*] NOS = not otherwise specified

[†] CNS = central nervous system

[‡] NHL = non-Hodgkin lymphoma

[§] GU = genitourinary

[‖] AML = acute myeloid leukaemia

## Supplemental Table 2: Cardiac disease classification: ICD revision 9 and ICD revision 10

| Cause of Death | ICD*-9 | ICD*-10 |
|---|---|---|
| **All cardiac disease** | **391, 392.0, 393-398, 402, 404, 410-414, 416, 420-429** | **I01, I02.0, I05-I09, I11, I13, I20-I25, I27.1-I27-9, I30-I52** |
| Cardiomyopathy/ congestive heart failure | 391.2, 398.0, 398.91, 402.01, 402.11, 402.91, 404.01, 404.03, 404.11, 404.13, 404.91, 404.93, 422, 425, 428, 429.0, 429.1, 429.3 | I01.2, I09.0, I11.0, I13.0, I13.2, I40-I43, I50, I51.4-5, I51.7 |
| Valvular heart disease | 424 | I34-I39 |
| Rheumatic valvular heart disease | 391.1, 394-397 | I01.1, I05-I08, I09.1 |
| Ischaemic heart disease | 410-414, 429.7 | I20-I25 |
| Arrhythmias | 426-427 | I44-49 |
| Pericardial disease | 391.0, 393, 420, 423 | I01.0, I09.2, I30-I32 |

* ICD = International Classification of Diseases

## Supplemental Table 3: Relative risks (RR) and excess mortality ratios (EMR) relating to age at cancer diagnosis and first primary cancer type from a multivariable Poisson regression model adjusted for the specified potential confounders [**,††] (corresponding to Table 2 and Table 3)

| Multivariable model | | All cardiac disease | | Ischaemic Heart Disease | | Valvular HD | | Cardiomyopathy / CHF [*] | |
|---|---|---|---|---|---|---|---|---|---|
| | | RR [†] (95% CI [‡]) | EMR [§] (95% CI) | RR (95% CI) | EMR (95% CI) | RR(95% CI) | EMR (95% CI) | RR (95% CI) | EMR (95% CI) |
| **Age at Cancer Diagnosis [**]** | 15-19 | **(ref)** [‖] | **(ref)** | **(ref)** | **(ref)** | **(ref)** | **(ref)** | **(ref)** | **(ref)** |
| | 20-24 | 0.7 (0.5,0.9) | 0.8 (0.5,1.1) | 0.8 (0.6,1.1) | 1.2 (0.8,2.0) | 0.5 (0.2,1.1) | 0.5 (0.2,1.2) | 0.7 (0.4,1.4) | 0.7 (0.2,2.3) |
| | 25-29 | 0.6 (0.4,0.7) | 0.7 (0.5,1.0) | 0.7 (0.5,0.9) | 1.1 (0.7,1.9) | 0.3 (0.1,0.7) | 0.3 (0.1,0.7) | 0.7 (0.4,1.4) | 0.6 (0.2,1.9) |
| | 30-34 | 0.5 (0.4,0.6) | 0.6 (0.4,0.9) | 0.6 (0.4,0.8) | 1.1 (0.6,1.9) | 0.2 (0.07,0.4) | 0.4 (0.0,0.2) | 0.9 (0.5,1.6) | 1.3 (0.4,3.6) |
| | 35-39 | 0.5 (0.4,0.6) | 0.6 (0.4,0.9) | 0.6 (0.4,0.8) | 1.2 (0.7,2.2) | 0.1 (0.1,0.3) | 0.2 (0.0,0.4) | 0.8 (0.4,1.5) | 0.8 (0.3,2.6) |
| | *2p for trend:* | *<0.0001* | *0.02* | *0.001* | *0.67* | *<0.0001* | *<0.0001* | *0.85* | *0.76* |
| **First Primary Cancer [††]** | Breast | 0.3 (0.2,0.3) | 0.1 (0.0,0.2) | 0.3 (0.2,0.4) | 0.1 (0.05,0.2) | 0.1 (0.05,0.3) | - | 0.2 (0.1,0.4) | 0.0 (0.0,0.5) |
| | Testicular | 0.3 (0.3,0.4) | 0.0 (0.0,0.5) | 0.3 (0.3,0.4) | - | 0.3 (0.1,0.6) | 0.2 (0.09,0.6) | 0.4 (0.2,0.6) | - |
| | Cervix | 0.3 (0.2,0.3) | 0.06 (0.0,0.2) | 0.3 (0.3,0.4) | 0.1 (0.05,0.2) | 0.1 (0.06,0.3) | - | 0.2 (0.1,0.3) | - |
| | Melanoma | 0.1 (0.1,0.2) | - [‡‡] | 0.1 (0.09,0.2) | - | 0.07 (0.02,0.2) | - | 0.2 (0.1,0.4) | - |
| | Central Nervous System Tumours | 0.4 (0.3,0.4) | 0.1 (0.1,0.3) | 0.4 (0.3,0.5) | 0.1 (0.07,0.3) | 0.2 (0.08,0.5) | 0.1 (0.0,0.5) | 0.4 (0.2,0.7) | 0.2 (0.1,0.7) |
| | Hodgkin | **(ref)** | **(ref)** | **(ref)** | **(ref)** | **(ref)** | **(ref)** | **(ref)** | **(ref)** |
| | Non-Hodgkin Lymphoma | 0.5 (0.4,0.6) | 0.3 (0.2,0.5) | 0.5 (0.4,0.6) | 0.3 (0.2,0.5) | 0.2 (0.08,0.7) | 0.2 (0.04,0.8) | 0.8 (0.5,1.4) | 0.6 (0.2,1.5) |
| | Thyroid | 0.2 (0.2,0.3) | 0.0 (0.0,0.4) | 0.2 (0.2,0.3) | - | 0.2 (0.09,0.7) | 0.2 (0.03,0.9) | 0.3 (0.1,0.7) | 0.1 (0.01,1.2) |
| | Gastrointestinal | 0.3 (0.2,0.3) | - | 0.3 (0.2,0.4) | - | 0.2 (0.05,0.5) | - | 0.4 (0.2,0.9) | - |
| | Soft Tissue Sarcoma | 0.3 (0.2,0.4) | 0.1 (0.0,0.3) | 0.3 (0.2,0.4) | - | 0.1 (0.03,0.5) | 0.1 (0.01,0.8) | 0.8 (0.4,1.5) | 0.7 (0.3,1.8) |
| | Ovary | 0.2 (0.1,0.3) | - | 0.2 (0.1,0.3) | - | 0.1 (0.03,0.6) | - | 0.4 (0.2,1.0) | 0.2 (0.0,1.2) |
| | Bladder | 0.3 (0.3,0.4) | 0.0 (0.0,0.4) | 0.3 (0.3,0.4) | - | 0.2 (0.05,0.6) | 0.1 (0.002,3.4) | 0.2 (0.1,0.6) | 0.1 (0.0,2.3) |
| | Kidney and GU tract [#] | 0.6 (0.4,0.7) | 0.5 (0.3,0.8) | 0.6 (0.5,0.8) | 0.6 (0.4,0.9) | 0.4 (0.1,1.0) | 0.3 (0.09,1.4) | 0.5 (0.2,1.0) | 0.3 (0.1,1.3) |
| | Head & Neck | 0.3 (0.3,0.5) | 0.1 (0.0,0.6] | 0.4 (0.3,0.5) | 0.0 (0.0,546.3) | 0.1 (0.01,0.7) | 0.0 (0.0,484.6) | 0.3 (0.1,0.9) | 0.2 (0.0,1.7) |
| | Leukaemia (excl. AML) [##] | 0.4 (0.3,0.6) | 0.2 (0.1,0.6) | 0.4 (0.3,0.7) | 0.2 (0.1,0.7) | 0 | - | 0.5 (0.1,1.5) | 0.3 (0.0,3.0) |
| | Other | 0.4 (0.3,0.6) | 0.2 (0.1,0.5) | 0.4 (0.3,0.6) | 0.2 (0.1,0.6) | 0.2 (0.05,1.0) | 0.2 (0.02,1.3) | 0.5 (0.2,1.4) | 0.5 (0.1,2.0) |
| | Bone Tumour | 0.3 (0.2,0.5) | 0.1 (0.0,0.6) | 0.3 (0.2,0.6) | 0.1 (0.0,1.1) | 0.2 (0.03,1.5) | - | 0.4 (0.09,1.6) | 0.1 (0.0,78.2) |
| | Acute Myeloid Leukaemia | 0.7 (0.4,1.1) | 0.5 (0.2,1.2) | 0.5 (0.2,1.0) | 0.3 (0.0,1.5) | 0.6 (0.08,4.5) | 0.6 (0.06,6.1) | 2.1 (0.9,4.9) | 1.9 (0.7,5.6) |
| | Lung | 0.5 (0.4,0.8) | 0.5 (0.3,1.0) | 0.5 (0.4,0.8) | 0.4 (0.2,1.1 | 0.5 (0.1,2.3) | 0.7 (0.1,3.3) | 0.9 (0.3,2.5) | 1.1 (0.3,4.2) |
| | *2p for heterogeneity:* | *<0.0001* | - | *<0.0001* | - | *<0.0001* | - | *<0.0001* | - |

[*] CHF = congestive heart failure
[†] RR = relative risks – can be interpreted as ratios of standardised mortality ratios adjusted for confounding risk factors included in the model
[‡] CI = confidence interval
[§] EMR = excess mortality ratio - can be interpreted as ratios of absolute excess risks adjusted for confounding risk factors included in the model
[‖] ref = reference group

[#] GU = genitourinary
[##] AML = acute myeloid leukaemia
[**] adjusted for gender, decade of cancer diagnosis, first primary cancer type and attained age
[††] adjusted for gender, age at cancer diagnosis, decade of cancer diagnosis and attained age
[‡‡] unreliable model fit due to small numbers of events

# Supplemental Table 4: Relative risks (RR) and excess mortality ratios (EMR) after specific cancers in relation to gender, age at cancer diagnosis, decade of cancer diagnosis and attained age from a multivariable Poisson regression model adjusted for the specified confounders [##,**,††,‡‡] (corresponding to Table 4)

| Multivariable model | Hodgkin lymphoma | | Non-Hodgkin lymphoma | | Central Nervous System Tumours | | Cervical Cancer | | Breast cancer | |
|---|---|---|---|---|---|---|---|---|---|---|
| | **RR [*] (95% CI [†])** | **EMR [‡] (95% CI)** | **RR (95% CI)** | **EMR (95% CI)** | **RR (95% CI)** | **EMR (95% CI)** | **RR (95% CI)** | **EMR (95% CI)** | **RR (95% CI)** | **EMR (95% CI)** |
| **Gender [##]** | | | | | | | | | | |
| Male | **(ref) [§]** | **(ref)** | **(ref)** | **(ref)** | **(ref)** | **(ref)** | - | - | - | - |
| Female | 1.7 (1.4,2.1) | 0.5 (0.4,0.7) | 1.8 (1.2,2.7) | 0.9 (0.4,1.9) | 1.3 (0.9,1.9) | 0.5 (0.2,1.5) | - | - | - | - |
| *2p for het* | *<0.0001* | *<0.0001* | *0.007* | *0.74* | *0.11* | *0.21* | - | - | - | - |
| **Age at Cancer Diagnosis [**]** | | | | | | | | | | |
| 15-19 | **(ref)** | **(ref)** | **(ref)** | **(ref)** | **(ref)** | **(ref)** | | | - | - |
| 20-24 | 0.6 (0.4,0.8) | 0.7 (0.5,1.0) | 2.3 (0.5,10.8) | -[#] | 0.9 (0.3,2.5) | 0.8 (0.0,13.2) | - | - | (ref) | (ref) |
| 25-29 | 0.4 (0.3,0.5) | 0.5 (0.3,0.7) | 1.9 (0.4,8.5) | - | 1.2 (0.5,3.0) | 3.2 (0.5,22.9) | (ref) | (ref) | 0.4 (0.1,1.4) | 0.4 (0.1,2.2) |
| 30-34 | 0.3 (0.2,0.5) | 0.5 (0.3,0.8) | 2.2 (0.5,9.3) | - | 0.8 (0.3,2.1) | 1.3 (0.1,25.0) | 1.9 (1.0,3.6) | - | 0.3 (0.1,1.0) | 0.1 (0.0,1.0) |
| 35-39 | 0.3 (0.2,0.4) | 0.6 (0.4,0.9) | 1.5 (0.3,6.5) | - | 1.0 (0.4,2.5) | 3.2 (0.2,43.2) | 1.7 (0.9,3.4) | - | 0.3 (0.1,0.8) | 0.1 (0.0,0.8) |
| *2p for trend* | *<0.0001* | *0.01* | *0.40* | - | *0.89* | *0.25* | *0.30* | - | *0.08* | *0.06* |
| **Decade of Cancer Diagnosis [††]** | | | | | | | | | | |
| 1970-79 | **(ref)** | **(ref)** | **(ref)** | **(ref)** | **(ref)** | **(ref)** | **(ref)** | **(ref)** | **(ref)** | **(ref)** |
| 1980-89 | 0.7 (0.6,0.9) | 0.4 (0.3,0.6) | 1.0 (0.6,1.5) | 0.5 (0.2,1.5) | 1.3 (0.9,1.9) | 2.3 (0.3,18.9) | 1.0 (0.7,1.4) | 1.2 (0.2,7.1) | 1.5 (1.1,2.0) | 1.9 (0.5,6.8) |
| 1990-99 | 0.8 (0.6,1.1) | 0.4 (0.2,0.6) | 1.2 (0.7,2.0) | 0.7 (0.3,1.9) | 1.3 (0.8,2.2) | 1.4 (0.1,14.4) | 0.8 (0.5,1.5) | 0.4 (0.0,4.4) | 0.9 (0.5,1.4) | - |
| 2000+ | 1.1 (0.6,2.1) | 0.4 (0.2,0.9) | 0.6 (0.2,1.9) | 0.1 (0.0,2.8) | 1.3 (0.5,3.3) | 1.3 (0.1,18.5) | 0.6 (0.1,2.4) | - | 0.6 (0.2,1.6) | 0.2 (0.0,37.8) |
| *2p for trend* | *0.11* | *<0.0001* | *0.95* | *0.15* | *0.27* | *0.93* | *0.48* | - | *0.87* | - |
| **Attained Age [‡‡]** | | | | | | | | | | |
| 20-39 | **(ref)** | **(ref)** | **(ref)** | **(ref)** | **(ref)** | **(ref)** | **(ref)** | **(ref)** | **(ref)** | **(ref)** |
| 40-49 | 1.0 (0.7,1.3) | 3.6 (2.4,5.5) | 0.8 (0.4,1.7) | 2.2 (0.6,7.6) | 0.5 (0.3,1.0) | 0.7 (0.1,4.3) | 0.5 (0.2,1.4) | 0.8 (0.1,5.0) | 0.6 (0.2,1.7) | 2.1 (0.3,14.7) |
| 50-59 | 0.9 (0.6,1.2) | 6.6 (4.2,10.3) | 0.6 (0.2,1.2) | 1.9 (0.4,9.1) | 0.5 (0.3,1.0) | 1.4 (0.2,9.4) | 0.4 (0.2,1.1) | 0.8 (0.1,6.5) | 0.4 (0.2,1.2) | 0.3 (0.0,245.4) |
| 60+ | 0.8 (0.6,1.2) | 12.5 (7.4,21.1) | 0.6 (0.2,1.4) | 6.4 (1.4,29.6) | 0.4 (0.2,0.9) | 1.6 (0.1,25.5) | 0.4 (0.1,1.0) | 1.1 (0.1,17.7) | 0.5 (0.2,1.3) | 5.4 (0.6,46.4) |
| *2p for trend* | *0.24* | *<0.0001* | *0.19* | *0.05* | *0.11* | *0.56* | *0.08* | *0.99* | *0.20* | *0.58* |

[*] RR = relative risks – can be interpreted as ratios of standardised mortality ratios adjusted for confounding risk factors included in the model
[†] CI = confidence interval
[‡] EMR = excess mortality ratio – can be interpreted as ratios of absolute excess risks adjusted for confounding risk factors included in the model
[§] ref = reference group

[#] unreliable model fit due to small numbers of events
[##] adjusted for age at cancer diagnosis, decade of cancer diagnosis, first primary cancer type and attained age
[**] adjusted for gender, decade of cancer diagnosis, first primary cancer type and attained age
[††] adjusted for gender, age at cancer diagnosis, first primary cancer type and attained age
[‡‡] adjusted for gender, age at cancer diagnosis, decade of cancer diagnosis and first primary cancer type

**Supplemental Table 5: Total excess cardiac deaths as a proportion of total excess deaths for all cancers combined and specific first primary cancers subdivided by attained age**

| | AER [*] cardiac / AER all causes (%) by attained age | | | | | | | | Total AER (%) | |
|---|---|---|---|---|---|---|---|---|---|---|
| | 20-39 | | 40-49 | | 50-59 | | 60+ | | | |
| Hodgkin Lymphoma | 3.9/75.9 | (5.2%) | 12.0/75.3 | (15.9%) | 24.9/118/3 | (21.0%) | 52.8/191.9 | (27.5%) | 12.9/89.0 | (14.5%) |
| Non-Hodgkin Lymphoma | 1.6/76.6 | (2.1%) | 4.2/130.8 | (3.2%) | 5.0/105.6 | (4.7%) | 14.4/101.5 | (14.2%) | 4.4/107.2 | (4.1%) |
| Central Nervous System Tumours | 1.3/181.8 | (0.7%) | 1.2/162.2 | (0.8%) | 2.6/95.8 | (2.7%) | 0.7/83.5 | (0.8%) | 1.5/150.4 | (1.0%) |
| Cervical Cancer | 0.4/38.9 | (1.0%) | 0.6/37.0 | (1.5%) | 1.0/33.8 | (3.0%) | 2.1/65.6 | (3.1%) | 0.8/39.7 | (2.1%) |
| Breast Cancer | 1.2/359.6 | (0.3%) | 0.7/258.5 | (0.3%) | 0.5/130.4 | (0.3%) | 2.4/86.4 | (2.8%) | 0.9/205.8 | (0.4%) |
| **All Cancers Combined** | **1.3/91.5** | **(1.4%)** | **1.9/103.5** | **(1.8%)** | **2.2/67.1** | **(3.3%)** | **2.9/66.8** | **(4.3%)** | **1.9/88.3** | **(2.2%)** |

[*] AER = absolute excess risk

## Supplemental Reference

1. Birch JM, Alston RD, Kelsey AM, Quinn MJ, Babb P, McNally RJQ. Classification and incidence of cancers in adolescents and young adults in England 1979-1997. *Br J Cancer*. 2002; 87: 1267-74.
